# Association Between the Preoperative Modified Frailty Index-5 and Cumulative Morbidity After Colorectal Cancer Resection: A Single-Center Retrospective Cohort Study Based on the Comprehensive Complication Index

**DOI:** 10.3390/jcm15208018

**Published:** 2026-10-16

**Authors:** İshak Aydın, Burak Aydoğan, Orçun Yalav, İbrahim Çoğal, Mevlüt Harun Ağca, Serdar Gümüş, Uğur Topal, Ahmet Gökhan Sarıtaş, İsmail Cem Eray

**Affiliations:** 1 Department of General Surgery, Cukurova University, 01330 Adana, Türkiye; 2 Department of General Surgery, Islahiye State Hospital, 27800 Gaziantep, Türkiye; 3 Department of Surgical Oncology, Cukurova University, 01330 Adana, Türkiye

**Keywords:** colorectal cancer, frailty, 5-factor modified frailty index, comprehensive complication index, postoperative morbidity

## Abstract

**Objective:** This study evaluated the association between the preoperative 5-factor Modified Frailty Index (mFI-5) and in-hospital cumulative postoperative morbidity, as measured by the Comprehensive Complication Index (CCI), in patients undergoing colorectal cancer resection. **Methods:** This retrospective, single-center cohort study included 455 patients who underwent resection for colorectal cancer between January 2015 and April 2021. Patients were classified as mFI-5 = 0, mFI-5 = 1, or mFI-5 ≥ 2. The primary outcome was high cumulative morbidity during the index hospitalization, defined as CCI ≥ 26.2. Associations with categorical outcomes were evaluated using multivariable logistic regression. Deviation from a linear trend was assessed using a likelihood ratio test, and sensitivity analyses were performed after excluding patients who died, restricting the analysis to elective patients, and using Firth penalized logistic regression. An exploratory interaction between categorical mFI-5 and emergency surgery was assessed using Firth penalized logistic regression. **Results:** Of the patients, 230 (50.5%) had mFI-5 = 0, 96 (21.1%) had mFI-5 = 1, and 129 (28.4%) had mFI-5 ≥ 2. The rates of CCI ≥ 26.2 were 17.8%, 12.5%, and 28.7%, respectively, and did not demonstrate a linear gradient across categories. Compared with mFI-5 = 0, mFI-5 ≥ 2 was associated with higher odds of CCI ≥ 26.2 after adjustment (OR 2.17, 95% CI 1.21–3.89; *p* = 0.010), whereas no association was observed for mFI-5 = 1 (OR 0.71, 95% CI 0.34–1.48; *p* = 0.364). A deviation from the linear trend was observed for CCI ≥ 26.2 (*p* = 0.025). In exploratory analyses, the adjusted OR was 2.43 (95% CI 1.30–4.54) among in-hospital survivors and 1.79 (95% CI 0.96–3.33) in the elective-only cohort. Firth analyses yielded similar results. The joint interaction test yielded *p* = 0.012, although the emergency subgroup contained a zero-event cell. **Conclusions:** mFI-5 ≥ 2 was associated with higher adjusted odds of high cumulative postoperative morbidity, whereas mFI-5 = 1 did not show an intermediate increase in risk. The non-monotonic pattern and attenuation of the association in the elective-only cohort support cautious interpretation of mFI-5 ≥ 2 as an exploratory risk indicator requiring external validation.

## 1. Introduction

Postoperative morbidity following colorectal cancer resection may manifest not merely as a single complication, but rather as the cumulative spectrum of clinical events occurring throughout hospitalization. Anastomotic leakage, intra-abdominal abscess, evisceration, events requiring reoperation, and postoperative death represent events at the severe end of the morbidity spectrum according to the Clavien–Dindo classification [1]. These events may prolong in-hospital recovery and may be accompanied by delays in the timing of adjuvant treatment [2]. In this context, not only the highest complication grade but also the total in-hospital burden of postoperative events accumulated within the same patient may be considered a distinct morbidity outcome.

Patient-based indicators such as age, ASA class, comorbidity, and functional status are used in preoperative clinical risk assessment for colorectal cancer surgery [3,4]. Frailty screening measures have been evaluated in various forms in the colorectal cancer surgery literature to assess this domain of patient-based vulnerability [4]. The 5-factor Modified Frailty Index (mFI-5) is one of the tools used in this setting as a concise measure of frailty/comorbidity burden derived from routinely available preoperative information [3,5]. In these studies, short-term surgical outcomes have most commonly been reported as separate endpoints, including the presence of postoperative complications, Clavien–Dindo classification, 30-day mortality, length of hospital stay, readmission, or reoperation [4,5].

Although the Clavien–Dindo classification is widely used for reporting postoperative complications, studies often focus on the most severe complication grade experienced by the patient [1,6]. Slankamenac et al. reported that this approach may fail to account for lower-grade complications and the total number of complications occurring in the same patient [6]. The Comprehensive Complication Index (CCI) was developed as a measure that integrates all postoperative complications, together with their severity, into a single patient-level score [6]. In a clinical validation study specifically conducted in colon cancer surgery, the CCI was compared with the Clavien–Dindo classification and used to assess the burden of postoperative morbidity [7].

Although studies in colorectal cancer surgery have reported frailty and the CCI together, the CCI in these studies has been used as a morbidity measure accompanying postoperative complication grading or long-term survival analyses [8]. In mFI-5-focused colorectal cancer studies, however, the CCI has not been positioned as the primary cumulative morbidity outcome calculated during the index hospitalization [5,9,10]. Therefore, the association between mFI-5 and the CCI calculated throughout the index hospitalization provides a framework in which patient-based vulnerability and cumulative postoperative morbidity are evaluated at the same outcome level.

The aim of this study was to evaluate the association between preoperative mFI-5 and cumulative postoperative morbidity, as measured by the CCI throughout the index hospitalization, in patients undergoing colorectal cancer resection. The secondary aim was to examine the association of mFI-5 with major postoperative morbidity and short-term surgical outcomes.

## 2. Material and Methods

### 2.1. Study Design and Population

**Figure 1:**
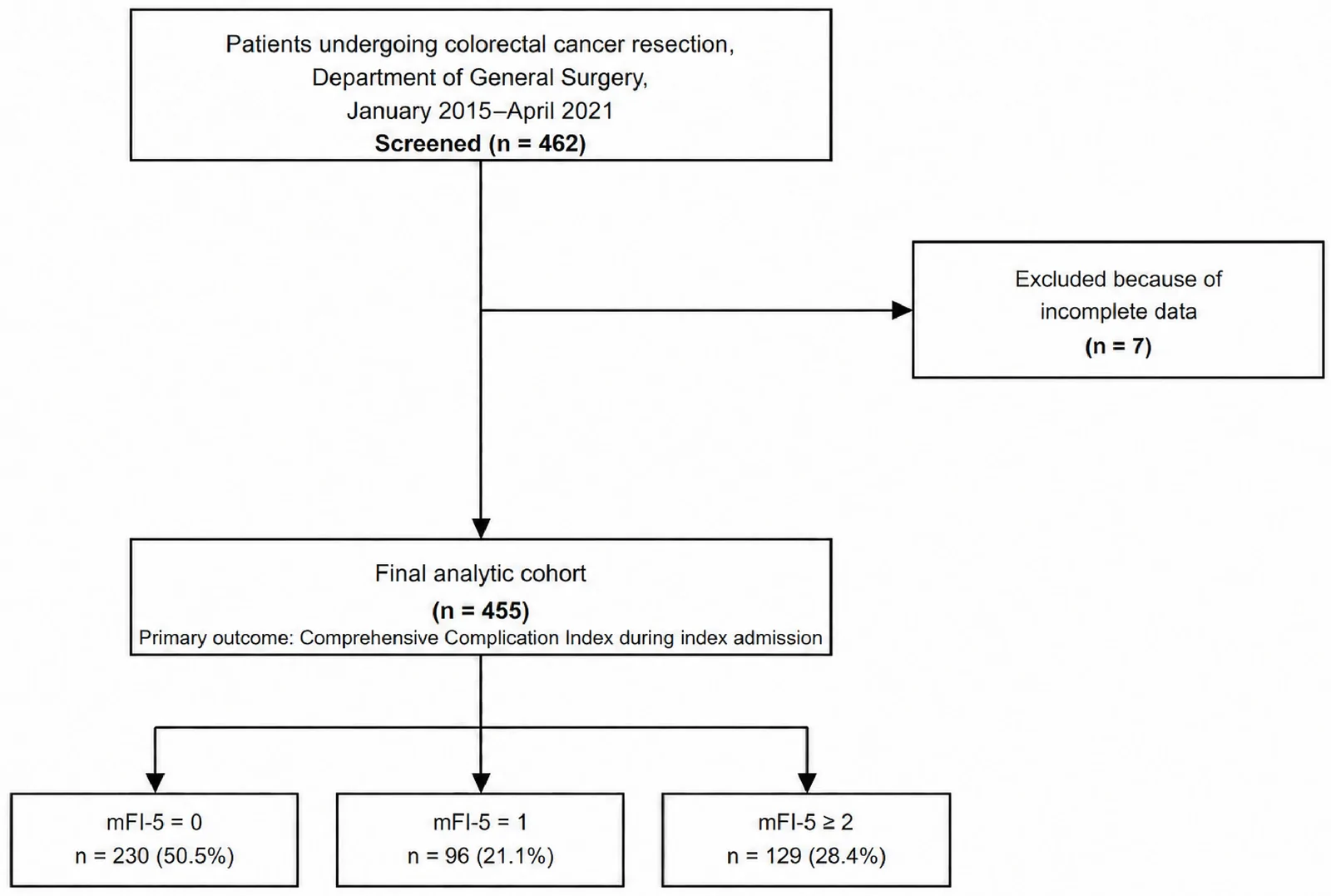
Patient flow diagram.

This retrospective single-center cohort study was conducted in the Department of General Surgery, Çukurova University Faculty of Medicine. Patients who underwent colorectal cancer resection between January 2015 and April 2021 were screened for eligibility. Patients with available preoperative clinical data, operative records, pathological data, and in-hospital postoperative complication records were included. Patients with incomplete data required for mFI-5 calculation or postoperative morbidity assessment were excluded. The final cohort consisted of 455 patients. The primary analysis included both elective and emergency resections to evaluate the association between preoperative mFI-5 and postoperative morbidity across the colorectal cancer resection population treated at our institution. Emergency surgery was included as a covariate in the primary adjusted models, and an elective-only sensitivity analysis was performed to assess the association after excluding emergency resections. The patient flow diagram is provided in Figure 1.

The study was approved by the Ethics Committee of Çukurova University Faculty of Medicine (approval no. 26; 10 July 2026). Research-related screening of historical records and data extraction commenced after ethics approval. The January 2015–April 2021 interval refers to the dates of colorectal cancer resection, not the timing of research-related record review. Because of the retrospective design, the requirement for informed consent was waived.

### 2.2. Data Collection and Variables

Demographic, clinical, operative, pathological, and postoperative data were obtained from institutional records. Baseline variables included age, sex, body mass index, ASA score, hemoglobin, albumin, neoadjuvant therapy, emergency surgery, tumor location, surgical approach, conversion to open surgery, stoma creation, additional organ resection, operation duration, and pathological stage. Pathological stage was analyzed as stage III–IV versus stage 0–II in regression models.

### 2.3. Definition of mFI-5

The modified frailty index-5 was calculated using five preoperative variables: hypertension requiring treatment, diabetes mellitus, chronic obstructive pulmonary disease, congestive heart failure, and dependent functional status [3]. Each variable was assigned 1 point, yielding a total score from 0 to 5. For descriptive and primary regression analyses, patients were categorized as mFI-5 = 0, mFI-5 = 1, and mFI-5 ≥ 2, with mFI-5 = 0 used as the reference category. The continuous mFI-5 score was evaluated in secondary analyses per 1-point increase.

### 2.4. Outcomes and Complication Assessment

The primary outcome was high cumulative postoperative morbidity during the index hospital stay, defined as CCI ≥ 26.2. Postoperative complications were retrospectively ascertained through review of discharge summaries, daily physician notes, nursing observations, medication records, laboratory results, and imaging reports from the index hospitalization. Postoperative complications were graded according to the Clavien–Dindo classification [1]. Clavien–Dindo grades were assigned by one investigator and subsequently checked by a second investigator, rather than independently graded by two investigators. No disagreements regarding complication grading were identified during this review. CCI was calculated from all in-hospital postoperative complications and expressed on a scale from 0 to 100 [6]. The threshold of CCI ≥ 26.2 corresponds to the CCI value assigned to a single Clavien–Dindo grade IIIa complication. This threshold was selected to define a clinically interpretable level of cumulative morbidity that can be reached through either a single complication requiring intervention or the accumulation of multiple lower-grade complications. The primary analysis therefore focused on the occurrence of high cumulative morbidity, whereas CCI distributions and secondary analyses of morbidity severity among patients with CCI > 0 provided complementary information on the continuous outcome.

Secondary outcomes included any postoperative morbidity (CCI > 0), Clavien–Dindo grade ≥ III complications, in-hospital mortality, postoperative length of stay, readmission, ICU admission, wound infection, ileus, intra-abdominal abscess, anastomotic leakage, evisceration, and postoperative bleeding. ICU admission was analyzed as a postoperative care-utilization outcome.

### 2.5. Statistical Analysis

Continuous variables were presented as median [interquartile range], and categorical variables were presented as *n* (%). Comparisons across mFI-5 categories were performed using the Kruskal–Wallis test for continuous variables and the chi-square or Fisher exact test for categorical variables, as appropriate.

Unadjusted and adjusted logistic regression models were used to evaluate the association between mFI-5 categories and binary postoperative morbidity outcomes. mFI-5 = 0 was used as the reference category, and effect estimates were reported separately for mFI-5 = 1 and mFI-5 ≥ 2 as odds ratios with 95% confidence intervals. Primary adjusted models included age, sex, body mass index, albumin, emergency surgery, open surgical approach, additional organ resection, operation duration, and pathological stage III–IV. Covariates were selected a priori on clinical grounds, before model fitting, to account for patient characteristics, nutritional status, operative factors, and disease stage; selection was not based on univariable statistical significance. ASA class was evaluated in additional sensitivity models because it captures aspects of baseline systemic disease burden that overlap conceptually with the comorbidity and functional-status components of mFI-5. It was omitted from the primary model to limit adjustment for overlapping measures of baseline health status. Additional ASA-adjusted models were fitted to assess whether the estimated associations were sensitive to accounting for this broader measure of preoperative health status. To assess potential confounding by tumor location, the model for CCI ≥ 26.2 was additionally adjusted for rectal versus non-rectal tumor location as a sensitivity analysis. Continuous per-point mFI-5 models were considered secondary analyses. Analyses of secondary outcomes were considered exploratory and were interpreted separately from the primary analysis. Because of the limited number of events in some binary outcome models, Firth penalized logistic regression was performed as an additional sensitivity analysis to assess the robustness of conventional maximum-likelihood estimates. ASA-adjusted categorical models and continuous per-point analyses are reported in Supplementary Table S1, and Firth penalized estimates are reported in Supplementary Table S2. Departure from a linear trend across mFI-5 categories was assessed by comparing a model containing separate indicator variables for mFI-5 = 1 and mFI-5 ≥ 2 with an ordinal trend model in which the categories were coded as 0, 1, and 2, using the likelihood-ratio test. Adjusted regression was not performed for in-hospital mortality because of the low number of events.

Exploratory analyses of binary morbidity outcomes were performed after excluding patients who died during the index hospitalization, and sensitivity analyses were performed after restricting the cohort to elective colorectal cancer resections. The categorical mFI-5 specification used in the primary analysis was retained in these models. As an exploratory component-specific analysis, patients with mFI-5 = 1 were stratified according to the single component contributing to the score. Frequencies of CCI > 0, CCI ≥ 26.2, and Clavien–Dindo grade ≥ III complications were compared across component-defined subgroups using the two-sided Fisher–Freeman–Halton exact test. *p* values were calculated by full enumeration of tables with fixed margins, summing the probabilities of tables no more probable than the observed table. Because of small subgroup sizes, separate component-specific multivariable models were not fitted. Among patients with CCI > 0, linear regression of log-transformed CCI was used to evaluate postoperative morbidity severity, with estimates presented separately for mFI-5 = 1 and mFI-5 ≥ 2 relative to mFI-5 = 0. Effect estimates from log-transformed CCI models were presented as multiplicative ratios with 95% confidence intervals. Emergency surgery was omitted from elective-only models. An exploratory interaction analysis for CCI ≥ 26.2 was performed by adding interaction terms between categorical mFI-5 and emergency surgery to the primary covariate specification. Because no events occurred among emergency patients with mFI-5 = 0, Firth penalized logistic regression was used. The two interaction terms were jointly assessed using a penalized likelihood-ratio test with two degrees of freedom, comparing the unrestricted model with the same model fitted under the constraint that both interaction coefficients were zero. Statistical analyses were performed using Python (version 3.12.14) with NumPy (version 2.3.5) and SciPy (version 1.17.0); Firth penalized logistic regression models were fitted using custom Python code. For the primary analysis, a two-sided *p* value < 0.05 was considered statistically significant. *p* values from secondary, sensitivity, and interaction analyses were considered exploratory, and no adjustment for multiple comparisons was applied.

## 3. Results

### 3.1. Study Population and Baseline Characteristics

A total of 455 patients who underwent colorectal cancer resection were included in the final analysis. The mFI-5 score was 0 in 230 patients, 1 in 96 patients, and ≥ 2 in 129 patients; 225 patients (49.5%) had at least one preoperative mFI-5 component. Among the 96 patients with mFI-5 = 1, the sole component was hypertension in 61 patients, diabetes mellitus in 20, dependent functional status in 10, and chronic obstructive pulmonary disease in 5; no patient had isolated congestive heart failure. Among the 129 patients with mFI-5 ≥ 2, hypertension was present in 114 (88.4%), diabetes mellitus in 92 (71.3%), dependent functional status in 57 (44.2%), chronic obstructive pulmonary disease in 24 (18.6%), and congestive heart failure in 16 (12.4%). The most frequent component combination was hypertension and diabetes mellitus without other mFI-5 components, observed in 52 patients (40.3%). Scores of 2, 3, 4, and 5 were recorded in 90, 35, 2, and 2 patients, respectively.

Baseline and operative characteristics according to mFI-5 category are summarized in Table 1. Median age increased from 58 years in the mFI-5 = 0 group to 67 years in the mFI-5 ≥ 2 group, whereas the proportion of male patients decreased across categories. Median ASA score was 1 in the mFI-5 = 0 group and 2 in both the mFI-5 = 1 and mFI-5 ≥ 2 groups. Hemoglobin levels were lower in patients with mFI-5 ≥ 2. BMI differed statistically across groups, with median values of 25.0, 26.0, and 26.0 kg/m^2^ in the mFI-5 = 0, mFI-5 = 1, and mFI-5 ≥ 2 groups, respectively.

Most operative and tumor-related variables did not differ significantly across mFI-5 categories. Neoadjuvant therapy, emergency surgery, tumor location, surgical approach, conversion to open surgery, stoma creation, additional organ resection, operation duration, and pathological stage III–IV showed no statistically significant differences across groups.

### 3.2. Postoperative Cumulative Morbidity According to mFI-5 Category

Postoperative outcomes according to mFI-5 category are summarized in Table 2. The distribution of CCI differed across mFI-5 categories but did not follow a monotonic gradient. Patients with mFI-5 ≥ 2 had the highest rates of CCI ≥ 26.2 and Clavien–Dindo grade ≥ III complications, whereas the mFI-5 = 1 group had lower crude rates than the mFI-5 = 0 group. A similar non-monotonic pattern was observed for any postoperative morbidity.

**Figure 2:**
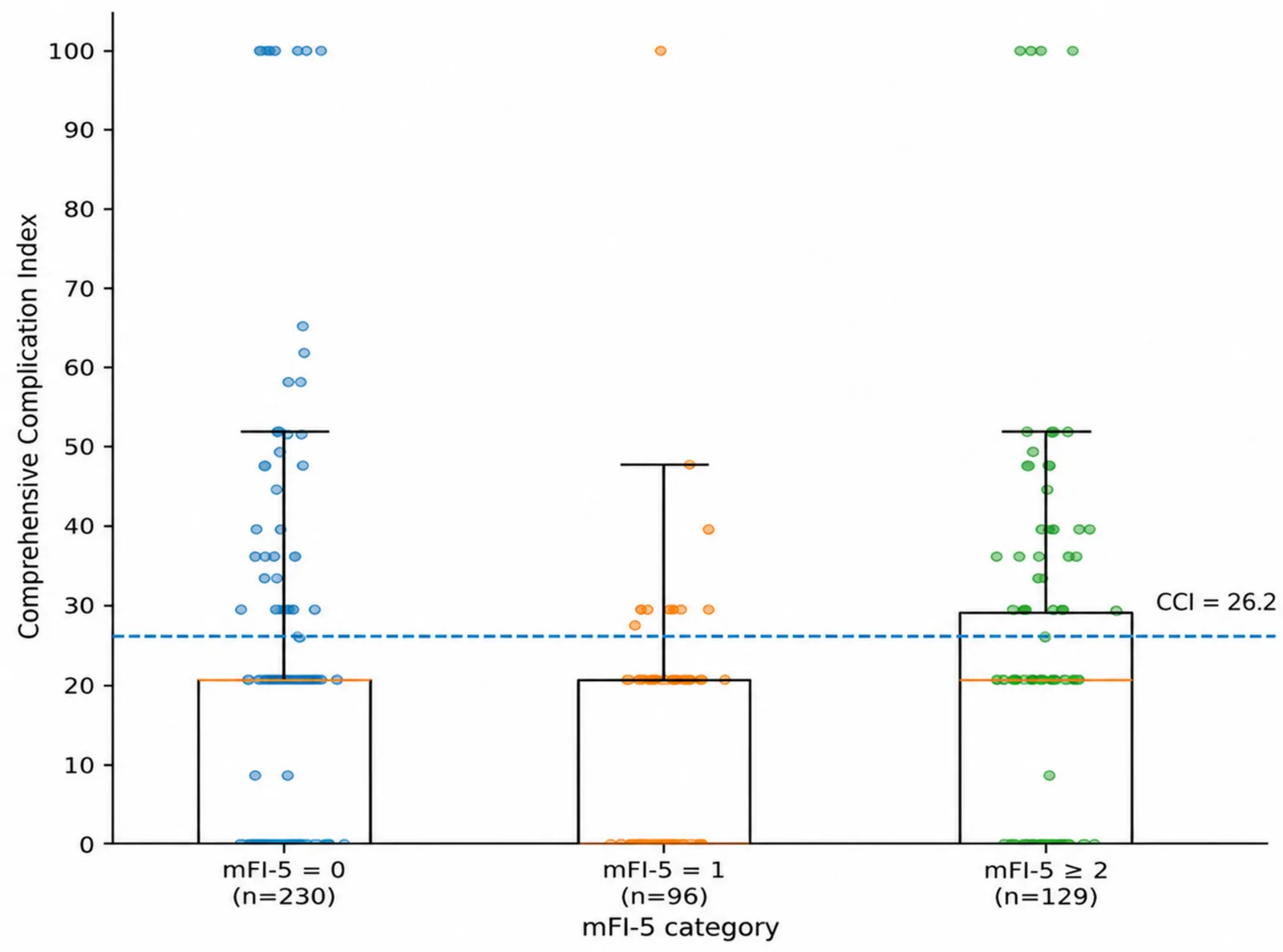
Distribution of Comprehensive Complication Index values according to mFI-5 category. Boxes indicate the median and interquartile range, whiskers indicate 1.5× interquartile range, and dots indicate individual patients. The dashed horizontal line indicates the CCI = 26.2 threshold for high cumulative morbidity. CCI, Comprehensive Complication Index; mFI-5, modified frailty index-5.

CCI ≥ 26.2 occurred in 28.7% of patients with mFI-5 ≥ 2, compared with 17.8% in patients with mFI-5 = 0 and 12.5% in patients with mFI-5 = 1. Clavien–Dindo grade ≥ III complications occurred in 18.6%, 11.7%, and 4.2% of these groups, respectively. In-hospital mortality did not differ significantly across mFI-5 categories. The distribution of CCI values across mFI-5 categories is shown in Figure 2.

### 3.3. Association Between mFI-5 and Postoperative Morbidity Outcomes

The associations between mFI-5 categories and postoperative morbidity outcomes are shown in Table 3. The crude outcome pattern was non-monotonic: compared with mFI-5 = 0, the mFI-5 = 1 group had lower odds of any postoperative morbidity and Clavien–Dindo grade ≥ III complications, whereas the mFI-5 ≥ 2 group had higher odds of CCI ≥ 26.2.

**Figure 3:**
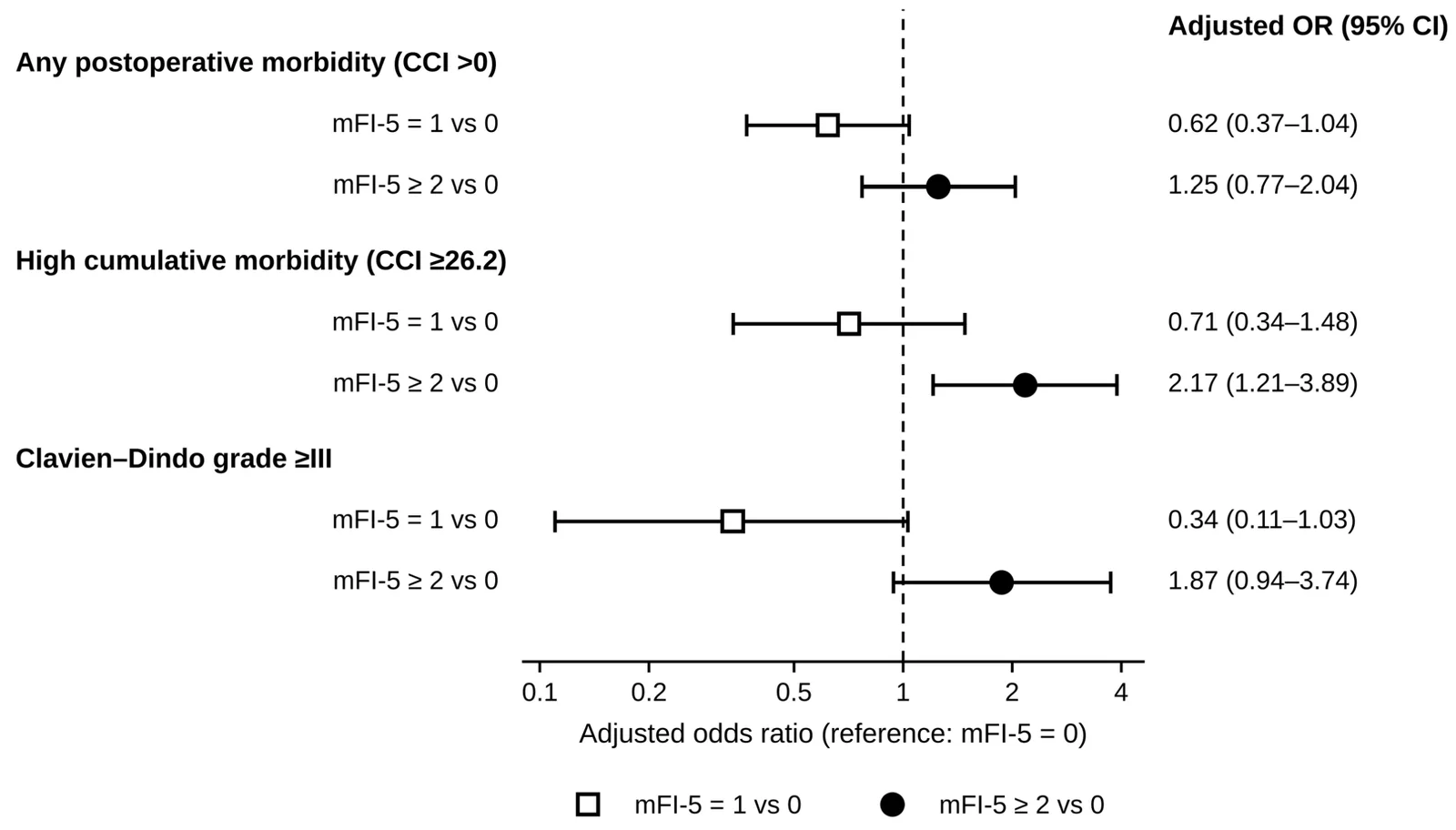
Adjusted associations between mFI-5 categories and binary postoperative morbidity outcomes. mFI-5 = 0 was used as the reference category. Open squares indicate estimates for mFI-5 = 1 versus 0, and filled circles indicate estimates for mFI-5 ≥ 2 versus 0; horizontal lines indicate 95% confidence intervals. Primary adjusted models included age, sex, body mass index, albumin, emergency surgery, open surgical approach, additional organ resection, operation duration, and pathological stage III–IV. The vertical dashed line indicates an odds ratio of 1. CCI, Comprehensive Complication Index; mFI-5, modified frailty index-5; OR, odds ratio.

After adjustment, mFI-5 ≥ 2 was associated with higher odds of CCI ≥ 26.2 compared with mFI-5 = 0. The adjusted associations of mFI-5 ≥ 2 with any postoperative morbidity and Clavien–Dindo grade ≥ III complications did not reach statistical significance. The mFI-5 = 1 group did not show an intermediate increase in risk. Likelihood-ratio tests indicated departure from a linear trend for CCI > 0 (*p* = 0.015), CCI ≥ 26.2 (*p* = 0.025), and Clavien–Dindo grade ≥ III complications (*p* = 0.002). Adjusted categorical estimates are shown in Figure 3. ASA-adjusted categorical models and continuous per-point analyses are provided in Supplementary Table S1. Firth penalized estimates were generally similar to the conventional maximum-likelihood estimates. For Clavien–Dindo grade ≥ III complications, the comparison of mFI-5 = 1 versus 0 yielded *p* = 0.048 with Firth regression, compared with *p* = 0.056 in the conventional model (Supplementary Table S2).

### 3.4. Sensitivity and Severity Analyses

Sensitivity and severity analyses are summarized in Table 4, and exploratory component-specific comparisons within the mFI-5 = 1 category are presented in Supplementary Table S3. Additional adjustment for rectal tumor location did not materially alter the primary association. Compared with mFI-5 = 0, mFI-5 ≥ 2 remained associated with higher odds of CCI ≥ 26.2 (OR 2.23, 95% CI 1.23–4.03; *p* = 0.008), whereas no association was observed for mFI-5 = 1 (OR 0.71, 95% CI 0.34–1.47; *p* = 0.357). After excluding patients who died during the index hospitalization, mFI-5 ≥ 2 remained associated with higher odds of CCI ≥ 26.2 and Clavien–Dindo grade ≥ III complications compared with mFI-5 = 0, whereas no statistically significant associations were observed for mFI-5 = 1.

In the elective-only cohort, the estimates for mFI-5 ≥ 2 were attenuated and did not reach statistical significance for either CCI ≥ 26.2 or Clavien–Dindo grade ≥ III complications. Compared with mFI-5 = 0, mFI-5 = 1 was not associated with CCI ≥ 26.2 but was associated with lower odds of Clavien–Dindo grade ≥ III complications. The exploratory Firth penalized interaction analysis identified evidence of interaction between categorical mFI-5 and emergency surgery for CCI ≥ 26.2 (joint penalized likelihood-ratio χ^2^ = 8.92, df = 2; *p* = 0.012). Among emergency resections, CCI ≥ 26.2 occurred in 0 of 24 patients with mFI-5 = 0, 3 of 13 with mFI-5 = 1, and 6 of 20 with mFI-5 ≥ 2. Corresponding event counts among elective resections were 41 of 206, 9 of 83, and 31 of 109, respectively.

Among patients with mFI-5 = 1, CCI ≥ 26.2 occurred in 7 of 61 patients (11.5%) with hypertension as the sole component, 3 of 20 (15.0%) with diabetes mellitus, 2 of 10 (20.0%) with dependent functional status, and 0 of 5 (0.0%) with chronic obstructive pulmonary disease. The exploratory comparison across component-defined subgroups yielded *p* = 0.769 for CCI ≥ 26.2, *p* = 0.106 for CCI > 0, and *p* = 0.626 for Clavien–Dindo grade ≥ III complications (Supplementary Table S3).

Among the 238 patients with CCI > 0, neither mFI-5 = 1 nor mFI-5 ≥ 2 was significantly associated with CCI severity compared with mFI-5 = 0 (adjusted ratios 0.91, 95% CI 0.77–1.08, and 1.10, 95% CI 0.95–1.28, respectively).

## 4. Discussion

In this study, the association between preoperative mFI-5 and cumulative morbidity, as measured by the CCI throughout the index hospitalization, did not demonstrate a linear score gradient. Compared with mFI-5 = 0, the mFI-5 ≥ 2 group had higher odds of CCI ≥ 26.2, whereas the mFI-5 = 1 group did not demonstrate an intermediate level of risk. For any postoperative morbidity and Clavien–Dindo ≥ III, individual categorical comparisons did not reach statistical significance after adjustment. Exploratory analyses among in-hospital survivors also showed associations of mFI-5 ≥ 2 with CCI ≥ 26.2 and Clavien–Dindo grade ≥ III complications, whereas the association with CCI ≥ 26.2 was attenuated in the elective-only cohort. This categorical pattern does not establish a biological or clinically validated threshold at mFI-5 ≥ 2.

Previous studies of colorectal cancer surgery have reported associations between mFI-5 and postoperative complications or severe postoperative morbidity [5,10,11]. Associations with other early postoperative outcomes, including length of hospital stay and short-term mortality, have also been described, although these findings have not been consistent across cohorts [5,9,10,11]. Within this literature, Keller et al. categorized patients as mFI-5 = 0, mFI-5 = 1, and mFI-5 ≥ 2 and reported that the association with major morbidity and mortality was concentrated in the mFI-5 ≥ 2 group [12]. The same study also demonstrated limited discriminatory performance of mFI-5 when used as a stand-alone predictive tool [12]. A recent systematic review and meta-analysis similarly found frailty to be associated with an increased risk of postoperative complications after colorectal cancer surgery; however, no statistically significant association with 30-day mortality was demonstrated, and substantial between-study heterogeneity was reported [13]. These studies provide context for the association observed in our cohort, although they do not establish that mFI-5 follows the same categorical risk pattern across populations.

The mFI-5 = 1 category comprised heterogeneous single-component profiles, predominantly hypertension alone. Each component contributes one point to the total mFI-5 score [3]. Thus, identical scores may represent different profiles of clinical vulnerability. Exploratory component-specific comparisons did not identify statistically significant differences in postoperative morbidity across these subgroups (Supplementary Table S3). However, several subgroups were small, limiting the precision of these comparisons and precluding conclusions regarding equivalent risk among components. Whether component composition contributed to the observed non-monotonic pattern therefore remains uncertain. The lower crude event rates in the mFI-5 = 1 group should not be interpreted as evidence of a protective association. Because the CCI accounts for both the number of complications occurring in the same patient and the severity of these events, it allows the association between mFI-5 and postoperative morbidity to be examined in terms of total complication burden rather than solely through the most severe complication grade [6]. Validation data from colon cancer surgery support the use of the CCI as a postoperative morbidity outcome [7]. In a study conducted after colorectal cancer surgery, the overall morbidity burden assessed using the CCI was also associated with readmission and long-term survival [14]. Accordingly, a distinct aspect of our study is the evaluation of mFI-5 not only in relation to major morbidity endpoints such as Clavien–Dindo ≥ III, but also within the same analytical framework as the cumulative morbidity burden accrued during the index hospitalization.

Associations between mFI-5 ≥ 2 and the binary morbidity outcomes remained apparent in analyses restricted to patients who survived the index hospitalization. However, conditioning on survival changes the population being evaluated and may introduce selection bias; these findings should therefore be interpreted as exploratory associations among in-hospital survivors rather than evidence of a mortality-independent association. In the elective-only analysis, the association between mFI-5 ≥ 2 and CCI ≥ 26.2 was attenuated, and its confidence interval included the null value. The formal interaction analysis provided exploratory evidence that the association differed by emergency surgery status on the odds-ratio scale. However, the emergency cohort included only 57 patients, with no CCI ≥ 26.2 events among the 24 patients with mFI-5 = 0. Although Firth penalization allowed estimation despite separation, the interaction finding remains sensitive to sparse data and requires confirmation in larger cohorts. Emergency colorectal cancer surgery has been associated with higher perioperative risk and increased need for intensive care [15]. The overall-cohort estimate should therefore not be assumed to represent a common association across elective and emergency resections. Among patients who developed complications, the severity analysis did not identify a statistically significant association between mFI-5 category and CCI severity. This analysis describes morbidity severity conditional on the occurrence of complications and should not be interpreted as an estimate of overall postoperative morbidity.

The mFI-5 captures a limited set of comorbidity and functional-status variables [3]. Patient- and procedure-related factors, including operative duration, surgical approach, and emergency presentation, have also been associated with short-term outcomes after colorectal cancer surgery [16]. In the present study, adjustment for age, albumin, and operative factors allowed the association between mFI-5 and cumulative morbidity to be evaluated while accounting for several relevant patient and procedural characteristics. However, covariate adjustment cannot fully separate the contribution of frailty-related vulnerability from other dimensions of baseline health status or eliminate residual confounding. The adjusted findings should therefore be interpreted as associations within the specified models rather than evidence that mFI-5 independently captures the full spectrum of perioperative vulnerability. Previous colorectal surgery studies have associated mFI-5 with length of hospital stay, short-term mortality, and early postoperative outcomes [9,17]. Higher mFI-5 categories have also been associated with Clavien–Dindo grade ≥ III complications and longer postoperative hospital stay in laparoscopic colorectal surgery [11]. In our cohort, postoperative length of stay did not differ significantly across categories, while the small number of in-hospital deaths limited mortality assessment. ICU admission represents postoperative care utilization and may reflect planned postoperative monitoring, institutional admission policies, and resource availability as well as complication severity. Accordingly, the between-group difference in ICU admission should not be interpreted as a corresponding difference in postoperative morbidity. These findings support further evaluation of mFI-5 as a potential patient-level indicator of cumulative postoperative morbidity. The absence of an intermediate risk increase in the mFI-5 = 1 category does not establish that scores of 0 and 1 are clinically interchangeable. Accordingly, the present findings do not justify replacing the three-category representation with a dichotomous classification of mFI-5 < 2 versus ≥ 2 in clinical practice. The potential utility of this threshold requires external validation and evaluation of predictive performance before clinical adoption. In the colorectal cancer surgery literature, frailty has been associated with adverse postoperative outcomes, although the strength and consistency of these associations have varied across outcomes and cohorts [4,13]. The ASCRS frailty guideline recommends incorporating frailty into the perioperative assessment and management of older patients undergoing colorectal surgery [18]. The ASCRS/SAGES ERAS guideline emphasizes multicomponent perioperative care principles in colorectal surgery [19]. The inclusion of patient-centered outcomes such as functional recovery, delirium, and quality of life in studies of older patients undergoing colorectal cancer surgery supports the view that risk assessment should not be limited to the presence of complications alone [20]. Whether mFI-5 adds useful information for perioperative counseling, optimization, monitoring, or discharge planning requires further evaluation.

This retrospective, single-center study is subject to potential selection bias, incomplete documentation, and center-specific practices, limiting generalizability. CCI assessment was restricted to the index hospitalization and did not capture post-discharge complications. Underdocumentation of Clavien–Dindo grades I–II may have underestimated cumulative morbidity; whether ascertainment differed across mFI-5 categories is unknown. Complication grades were checked by a second investigator, but interobserver reliability was not formally assessed. Temporal changes in perioperative practice during 2015–2021 may have introduced residual confounding. Although albumin was included in adjusted models, nutritional status was not comprehensively assessed, and other frailty domains and patient-centered outcomes were not evaluated. Limited event counts relative to model complexity may have reduced precision, and Firth penalization does not eliminate uncertainty arising from sparse data. This limitation particularly affects the interaction analysis, which included a zero-event cell in the emergency mFI-5 = 0 subgroup. Small component-defined subgroups and few patients with scores of 3–5 also limited component-specific inference and assessment of the score–outcome relationship. Analyses excluding deaths were conditional on survival and susceptible to selection bias; they cannot establish a mortality-independent association. Discrimination, calibration, and incremental predictive value were not assessed, so the findings do not establish predictive performance or clinical utility. In conclusion, mFI-5 ≥ 2 was associated with higher adjusted odds of high cumulative postoperative morbidity, defined as CCI ≥ 26.2, compared with mFI-5 = 0, whereas mFI-5 = 1 did not show an intermediate increase in risk. Attenuation in the elective-only cohort and the exploratory interaction finding limit interpretation of the overall estimate as a common association across surgical settings. The non-monotonic pattern, sparse subgroup data, and limited representation of higher scores preclude establishing a clinical cutoff. External validation is required before clinical adoption.

## Figures and Tables

**Table 1:** Baseline and operative characteristics according to mFI-5 category.

Variable	Overall *n* = 455	mFI-5 = 0 *n* = 230	mFI-5 = 1 *n* = 96	mFI-5 ≥ 2 *n* = 129	*p* Value
Age, years	62 [54–68]	58 [48–65]	64 [58–68]	67 [61–73]	<0.001
Male sex	286 (62.9)	164 (71.3)	53 (55.2)	69 (53.5)	<0.001
BMI, kg/m^2^	25.6 [23.8–28.0]	25.0 [23.5–27.6]	26.0 [24.1–29.6]	26.0 [23.9–29.2]	0.004
ASA score	2 [1–2]	1 [1–2]	2 [2–2]	2 [2–3]	<0.001
Hemoglobin, g/dL	11.8 [10.5–13.4]	12.3 [10.7–13.7]	11.8 [10.6–13.4]	11.2 [10.5–12.5]	0.004
Albumin, g/L	35.0 [30.4–37.4]	35.0 [32.0–38.0]	35.0 [29.1–36.6]	35.0 [30.0–37.2]	0.061
Neoadjuvant therapy	132 (29.0)	74 (32.2)	30 (31.2)	28 (21.7)	0.096
Emergency surgery	57 (12.5)	24 (10.4)	13 (13.5)	20 (15.5)	0.358
Tumor location					0.053
Right colon	135 (29.7)	62 (27.0)	27 (28.1)	46 (35.7)	
Left colon	149 (32.7)	69 (30.0)	32 (33.3)	48 (37.2)	
Rectum	159 (34.9)	93 (40.4)	36 (37.5)	30 (23.3)	
Other/multiple	12 (2.6)	6 (2.6)	1 (1.0)	5 (3.9)	
Open surgery	247 (54.3)	117 (50.9)	54 (56.2)	76 (58.9)	0.310
Conversion to open surgery	20 (4.4)	13 (5.7)	5 (5.2)	2 (1.6)	0.174
Stoma created	182 (40.0)	100 (43.5)	40 (41.7)	42 (32.6)	0.120
Additional organ resection	134 (29.5)	68 (29.6)	21 (21.9)	45 (34.9)	0.106
Operation duration, min	180 [150–200]	180 [150–200]	180 [150–196]	180 [150–200]	0.706
Pathologic stage III–IV	220 (48.4)	104 (45.2)	46 (47.9)	70 (54.3)	0.257

Values are presented as median [interquartile range] or *n* (%). mFI-5 was calculated using hypertension, diabetes mellitus, chronic obstructive pulmonary disease, congestive heart failure, and dependent functional status. For tumor location, the *p* value refers to the overall comparison across categories. *p* values were calculated using the Kruskal–Wallis test for continuous variables and the chi-square or Fisher exact test for categorical variables, as appropriate. ASA, American Society of Anesthesiologists; BMI, body mass index; mFI-5, modified frailty index-5.

**Table 2:** Postoperative morbidity and clinical outcomes according to mFI-5 category.

Variable	Overall *n* = 455	mFI-5 = 0 *n* = 230	mFI-5 = 1 *n* = 96	mFI-5 ≥ 2 *n* = 129	*p* Value
CCI	20.9 [0.0–20.9]	20.9 [0.0–20.9]	0.0 [0.0–20.9]	20.9 [0.0–29.6]	0.010
Any postoperative morbidity, CCI > 0	238 (52.3)	124 (53.9)	40 (41.7)	74 (57.4)	0.052
CCI ≥ 26.2	90 (19.8)	41 (17.8)	12 (12.5)	37 (28.7)	0.006
Clavien–Dindo grade ≥ III	55 (12.1)	27 (11.7)	4 (4.2)	24 (18.6)	0.004
In-hospital mortality	13 (2.9)	8 (3.5)	1 (1.0)	4 (3.1)	0.475
Postoperative length of stay, days	7 [6–11]	8 [6–11]	7 [5–11]	8 [5–11]	0.417
Readmission	60 (13.2)	27 (11.7)	10 (10.4)	23 (17.8)	0.174
Any ICU admission	189 (41.5)	73 (31.7)	53 (55.2)	63 (48.8)	<0.001
Wound infection	113 (24.8)	51 (22.2)	27 (28.1)	35 (27.1)	0.408
Ileus	45 (9.9)	23 (10.0)	8 (8.3)	14 (10.9)	0.819
Intra-abdominal abscess	30 (6.6)	17 (7.4)	2 (2.1)	11 (8.5)	0.123
Anastomotic leakage	13 (2.9)	5 (2.2)	1 (1.0)	7 (5.4)	0.101
Evisceration	14 (3.1)	8 (3.5)	1 (1.0)	5 (3.9)	0.420
Postoperative bleeding	9 (2.0)	4 (1.7)	0 (0.0)	5 (3.9)	0.111

Values are presented as median [interquartile range] or *n* (%). Comparisons were performed using the Kruskal–Wallis test for continuous variables and Pearson’s χ^2^ or Fisher’s exact test for categorical variables, as appropriate. CCI, Comprehensive Complication Index; ICU, intensive care unit; mFI-5, modified frailty index-5.

**Table 3:** Association between mFI-5 categories and postoperative morbidity outcomes.

Outcome	Contrast	Events/*N*	Unadjusted OR (95% CI)	*p* Value	Adjusted OR (95% CI)	*p* Value
Any postoperative morbidity, CCI > 0	mFI-5 = 1 vs. 0	238/455	0.61 (0.38–0.99)	0.045	0.62 (0.37–1.04)	0.071
	mFI-5 ≥ 2 vs. 0	238/455	1.15 (0.74–1.78)	0.528	1.25 (0.77–2.04)	0.373
High cumulative morbidity, CCI ≥ 26.2	mFI-5 = 1 vs. 0	90/455	0.66 (0.33–1.32)	0.237	0.71 (0.34–1.48)	0.364
	mFI-5 ≥ 2 vs. 0	90/455	1.85 (1.11–3.09)	0.018	2.17 (1.21–3.89)	0.010
Clavien–Dindo grade ≥ III	mFI-5 = 1 vs. 0	55/455	0.33 (0.11–0.96)	0.042	0.34 (0.11–1.03)	0.056
	mFI-5 ≥ 2 vs. 0	55/455	1.72 (0.94–3.13)	0.076	1.87 (0.94–3.74)	0.076

Values are odds ratios with 95% confidence intervals. mFI-5 = 0 was used as the reference category. Primary adjusted models included age, sex, body mass index, albumin, emergency surgery, open surgical approach, additional organ resection, operation duration, and pathological stage III–IV. ASA-adjusted categorical models and continuous per-point analyses are presented in Supplementary Table S1; Firth penalized estimates are presented in Supplementary Table S2. CCI, Comprehensive Complication Index; mFI-5, modified frailty index-5; OR, odds ratio.

**Table 4:** Sensitivity and severity analyses according to mFI-5 category.

Analysis	Outcome	Contrast	Events or Analysis Population	Adjusted Effect Estimate (95% CI)	*p* Value
Rectal-location adjusted model	CCI ≥ 26.2	mFI-5 = 1 vs. 0	90/455	OR 0.71 (0.34–1.47)	0.357
		mFI-5 ≥ 2 vs. 0	90/455	OR 2.23 (1.23–4.03)	0.008
Deaths excluded	CCI ≥ 26.2	mFI-5 = 1 vs. 0	77/442	OR 0.85 (0.40–1.84)	0.684
		mFI-5 ≥ 2 vs. 0	77/442	OR 2.43 (1.30–4.54)	0.005
Deaths excluded	Clavien–Dindo grade ≥ III	mFI-5 = 1 vs. 0	42/442	OR 0.40 (0.11–1.43)	0.157
		mFI-5 ≥ 2 vs. 0	42/442	OR 2.32 (1.06–5.04)	0.034
Elective-only cohort	CCI ≥ 26.2	mFI-5 = 1 vs. 0	81/398	OR 0.49 (0.22–1.10)	0.085
		mFI-5 ≥ 2 vs. 0	81/398	OR 1.79 (0.96–3.33)	0.068
Elective-only cohort	Clavien–Dindo grade ≥ III	mFI-5 = 1 vs. 0	50/398	OR 0.31 (0.10–0.96)	0.043
		mFI-5 ≥ 2 vs. 0	50/398	OR 1.42 (0.68–2.99)	0.351
Patients with CCI > 0	Log-transformed CCI severity	mFI-5 = 1 vs. 0	238 patients	Ratio 0.91 (0.77–1.08)	0.270
		mFI-5 ≥ 2 vs. 0	238 patients	Ratio 1.10 (0.95–1.28)	0.187

Values are adjusted effect estimates with 95% confidence intervals. mFI-5 = 0 was used as the reference category. Logistic regression was used for binary outcomes, and linear regression of log-transformed CCI was used for severity analyses among patients with CCI > 0. Ratios indicate the multiplicative difference in CCI severity relative to mFI-5 = 0. Primary adjusted models included age, sex, body mass index, albumin, emergency surgery, open surgical approach, additional organ resection, operation duration, and pathological stage III–IV. Emergency surgery was omitted from elective-only models. The rectal-location-adjusted sensitivity model additionally included rectal versus non-rectal tumor location. CCI, Comprehensive Complication Index; mFI-5, modified frailty index-5; OR, odds ratio.

## Data Availability

The data supporting the findings of this study are not publicly available because they contain confidential patient information. De-identified data may be made available from the corresponding authors upon reasonable request, subject to institutional approval and applicable ethical restrictions.

## Supplementary Materials

The following supporting information can be downloaded at https://www.mdpi.com/article/10.3390/jcm15208018/s1, Table S1: Secondary continuous and ASA-adjusted analyses of postoperative morbidity outcomes; Table S2: Firth penalized logistic regression sensitivity analyses for binary postoperative morbidity outcomes; Table S3: Exploratory postoperative morbidity outcomes according to the individual component defining mFI-5 = 1.

## References

[1] Dindo D., Demartines N., Clavien P.A. (2004). Classification of surgical complications: A new proposal with evaluation in a cohort of 6336 patients and results of a survey. Ann. Surg..

[2] Fransgaard T., Thygesen L.C., Gögenur I. (2021). The impact of postoperative complications and delay of adjuvant chemotherapy on oncological outcomes in patients with colorectal cancer. Colorectal Dis..

[3] Subramaniam S., Aalberg J.J., Soriano R.P., Divino C.M. (2018). New 5-factor modified frailty index using American College of Surgeons NSQIP data. J. Am. Coll. Surg..

[4] McGovern J., Dolan R.D., Horgan P.G., Laird B.J., McMillan D.C. (2022). The prevalence and prognostic value of frailty screening measures in patients undergoing surgery for colorectal cancer: Observations from a systematic review. BMC Geriatr..

[5] McGovern J., Grayston A., Coates D., Leadbitter S., Hounat A., Horgan P.G., Dolan R.D., McMillan D.C. (2023). The relationship between the modified frailty index score (mFI-5), malnutrition, body composition, systemic inflammation and short-term clinical outcomes in patients undergoing surgery for colorectal cancer. BMC Geriatr..

[6] Slankamenac K., Graf R., Barkun J., Puhan M.A., Clavien P.A. (2013). The Comprehensive Complication Index: A novel continuous scale to measure surgical morbidity. Ann. Surg..

[7] Tamini N., Bernasconi D., Ripamonti L., Lo Bianco G., Braga M., Nespoli L. (2021). Clinical validation of the Comprehensive Complication Index in colon cancer surgery. Cancers.

[8] Artiles-Armas M., Roque-Castellano C., Fariña-Castro R., Conde-Martel A., Acosta-Mérida M.A., Marchena-Gómez J. (2021). Impact of frailty on 5-year survival in patients older than 70 years undergoing colorectal surgery for cancer. World J. Surg. Oncol..

[9] Ogata T., Sadakari Y., Nakane H., Koikawa K., Kanno H., Kohata R., Endo K., Tsukahara T., Shimonaga K., Kaneshiro K. (2023). The five-item modified frailty index predicts long-term outcomes in elderly patients undergoing colorectal cancer surgery. World J. Surg. Oncol..

[10] Huang L., Li Z., Jian M., Wu X., Chen H., Qin H., Li Z., Song S., Xie Y., Chen R. (2023). Application of MFI-5 in severe complications and unfavorable outcomes after radical resection of colorectal cancer. World J. Surg. Oncol..

[11] Okada S., Inoue Y., Adachi T., Soyama A., Kobayashi K., Hidaka M., Kanetaka K., Eguchi S. (2024). Five-item modified frailty index in elderly patients undergoing laparoscopic colorectal surgery predicts postoperative complications. Cancer Diagn. Progn..

[12] Keller D.S., Reif de Paula T., Kiran R.P. (2020). Evaluating the association of the new National Surgical Quality Improvement Program modified 5-factor frailty index with outcomes in elective colorectal surgery. Color. Dis..

[13] Xia L., Yin R., Mao L., Shi X. (2024). Prevalence and impact of frailty in patients undergoing colorectal cancer surgery: A systematic review and meta-analysis based on modified frailty index. J. Surg. Oncol..

[14] Slankamenac K., Slankamenac M., Schlegel A., Nocito A., Rickenbacher A., Clavien P.-A., Turina M. (2017). Impact of postoperative complications on readmission and long-term survival in patients following surgery for colorectal cancer. Int. J. Colorectal Dis..

[15] Dale C., McLoone P., Sloan B., Kinsella J., Morrison D., Puxty K., Quasim T. (2016). Critical care provision after colorectal cancer surgery. BMC Anesthesiol..

[16] Moro-Valdezate D., Martín-Arévalo J., Ferro-Echevarría Ó., Pla-Martí V., García-Botello S., Pérez-Santiago L., Gadea-Mateo R., Tarazona N., Roda D., Roselló-Keränen S. (2022). Short-term outcomes of colorectal cancer surgery in older patients: A novel nomogram predicting postoperative morbi-mortality. Langenbecks Arch. Surg..

[17] Al-Khamis A., Warner C., Park J., Marecik S., Davis N., Mellgren A., Nordenstam J., Kochar K. (2019). Modified frailty index predicts early outcomes after colorectal surgery: An ACS-NSQIP study. Colorectal Dis..

[18] Saur N.M., Davis B.R., Montroni I., Shahrokni A., Rostoft S., Russell M.M., Mohile S.G., Suwanabol P.A., Lightner A.L., Poylin V. (2022). Clinical Practice Guidelines Committee of the American Society of Colon and Rectal Surgeons. The American Society of Colon and Rectal Surgeons clinical practice guidelines for the perioperative evaluation and management of frailty among older adults undergoing colorectal surgery. Dis. Colon. Rectum..

[19] Irani J., Hedrick T.L., Miller T.E., Lee L., Steinhagen E., Shogan B.D., Goldberg J.E., Feingold D.L., Lightner A.L., Paquette I.M. (2023). Clinical practice guidelines for enhanced recovery after colon and rectal surgery from the American Society of Colon and Rectal Surgeons and the Society of American Gastrointestinal and Endoscopic Surgeons. Dis. Colon. Rectum..

[20] Xavier H., Bhattacharya Y., Poobalan A., Brazzelli M., Ramsay G. (2024). Outcomes reported in elective colorectal cancer surgery research for older patients: A scoping review. Colorectal Dis..

