# Supplementary material for: Association Between the Preoperative Modified Frailty Index-5 and Cumulative Morbidity After Colorectal Cancer Resection: A Single-Center Retrospective Cohort Study Based on the Comprehensive Complication Index
Source: J Clin Med. 2026 Oct 16;15(20):8018. doi: 10.3390/jcm15208018 (PMC13706162; doi:10.3390/jcm15208018)
Supplement: Supplementary file 1 [file jcm-15-08018-s001.zip › jcm-4562924-supplementary.pdf]

Supplementary Table S1. Secondary continuous and ASA-adjusted analyses of postoperative morbidity outcomes

Panel A. Categorical models additionally adjusted for ASA class

Outcome	Parameter	Adjusted OR (95% CI)	p value
Any postoperative morbidity, CCI >0	mFI-5=1 vs 0	0.73 (0.42–1.27)	0.265
	mFI-5≥2 vs 0	1.69 (0.92–3.12)	0.091
	ASA class, per category	0.74 (0.51–1.06)	0.100
High cumulative morbidity, CCI ≥26.2	mFI-5=1 vs 0	1.05 (0.49–2.28)	0.899
	mFI-5≥2 vs 0	4.63 (2.11–10.15)	<0.001
	ASA class, per category	0.47 (0.28–0.78)	0.003
Clavien-Dindo grade ≥III	mFI-5=1 vs 0	0.59 (0.19–1.86)	0.367
	mFI-5≥2 vs 0	6.08 (2.27–16.32)	<0.001
	ASA class, per category	0.31 (0.16–0.62)	<0.001

Panel B. Continuous per-point mFI-5 analyses

Outcome	Unadjusted OR per 1-point increase (95% CI)	p value	Adjusted OR per 1-point increase (95% CI)	p value
Any postoperative morbidity, CCI >0	1.07 (0.90–1.27)	0.459	1.13 (0.92–1.37)	0.243
High cumulative morbidity, CCI ≥26.2	1.34 (1.09–1.65)	0.006	1.48 (1.16–1.88)	0.001
Clavien-Dindo grade ≥III	1.35 (1.06–1.73)	0.017	1.46 (1.10–1.94)	0.008

Values are odds ratios with 95% confidence intervals. In Panel A, mFI-5=0 was used as the reference category, and models included age, sex, body mass index, ASA class, albumin, emergency surgery, open surgical approach, additional organ resection, operation duration, and pathological stage III–IV. ASA estimates are

presented per 1-category increase. In Panel B, adjusted continuous models included the same covariates except ASA class. Continuous analyses were considered secondary because the primary categorical analyses indicated departure from linearity. ASA, American Society of Anesthesiologists; CCI, Comprehensive Complication Index; mFI-5, modified frailty index-5; OR, odds ratio.

Supplementary Table S2. Firth penalized logistic regression sensitivity analyses for binary postoperative morbidity outcomes

Analysis	Outcome	Contrast	Events / N	Conventional adjusted OR (95% CI)	Firth adjusted OR (95% CI)	Firth p value
Primary	CCI >0	mFI-5=1 vs 0	238 / 455	0.62 (0.37–1.04)	0.63 (0.38–1.05)	0.074
		mFI-5≥2 vs 0	238 / 455	1.25 (0.77–2.04)	1.24 (0.77–2.02)	0.379
Primary	CCI ≥26.2	mFI-5=1 vs 0	90 / 455	0.71 (0.34–1.48)	0.73 (0.35–1.46)	0.386
		mFI-5≥2 vs 0	90 / 455	2.17 (1.21–3.89)	2.12 (1.19–3.79)	0.010
Primary	Clavien-Dindo grade ≥III	mFI-5=1 vs 0	55 / 455	0.34 (0.11–1.03)	0.38 (0.12–0.99)	0.048
		mFI-5≥2 vs 0	55 / 455	1.87 (0.94–3.74)	1.84 (0.93–3.63)	0.079
Deaths excluded	CCI ≥26.2	mFI-5=1 vs 0	77 / 442	0.85 (0.40–1.84)	0.87 (0.40–1.81)	0.721
		mFI-5≥2 vs 0	77 / 442	2.43 (1.30–4.54)	2.37 (1.29–4.39)	0.006
Deaths excluded	Clavien-Dindo grade ≥III	mFI-5=1 vs 0	42 / 442	0.40 (0.11–1.43)	0.45 (0.11–1.35)	0.163
		mFI-5≥2 vs 0	42 / 442	2.32 (1.06–5.04)	2.24 (1.05–4.83)	0.036
Elective-only cohort	CCI ≥26.2	mFI-5=1 vs 0	81 / 398	0.49 (0.22–1.10)	0.51 (0.22–1.09)	0.086

		mFI-5≥2 vs 0	81 / 398	1.79 (0.96–3.33)	1.76 (0.95–3.25)	0.070
Elective-only cohort	Clavien-Dindo grade ≥III	mFI-5=1 vs 0	50 / 398	0.31 (0.10–0.96)	0.35 (0.11–0.94)	0.036
		mFI-5≥2 vs 0	50 / 398	1.42 (0.68–2.99)	1.42 (0.68–2.92)	0.347

Conventional estimates were obtained using maximum-likelihood logistic regression. Firth estimates were obtained using penalized logistic regression; 95% confidence intervals were derived from the profile penalized likelihood, and p values from penalized likelihood-ratio tests. Models included age, sex, body mass index, albumin, emergency surgery, open surgical approach, additional organ resection, operation duration, and pathological stage III–IV. Emergency surgery was omitted from elective-only models. mFI-5=0 was used as the reference category. CCI, Comprehensive Complication Index; mFI-5, modified frailty index-5; OR, odds ratio.

Supplementary Table S3. Exploratory postoperative morbidity outcomes according to the individual component defining mFI-5=1

Individual component defining mFI-5=1	n	CCI >0, n (%)	CCI ≥26.2, n (%)	Clavien–Dindo grade ≥III, n (%)
Hypertension	61	30 (49.2)	7 (11.5)	2 (3.3)
Diabetes mellitus	20	6 (30.0)	3 (15.0)	1 (5.0)
Dependent functional status	10	4 (40.0)	2 (20.0)	1 (10.0)
Chronic obstructive pulmonary disease	5	0 (0.0)	0 (0.0)	0 (0.0)
Exact p value	—	0.106	0.769	0.626

Only patients with mFI-5=1 were included in this exploratory analysis. Percentages were calculated within each component-defined subgroup. No patient had congestive heart failure as the sole mFI-5 component. Two-sided Fisher–Freeman–Halton exact tests were performed using full enumeration of tables with fixed margins and probability-based ordering. P values were considered exploratory, and no adjustment for multiple comparisons was applied. CCI, Comprehensive Complication Index; mFI-5, modified frailty index-5.
